# Genetic Variation of Human Papillomavirus Type 16 in Individual Clinical Specimens Revealed by Deep Sequencing

**DOI:** 10.1371/journal.pone.0080583

**Published:** 2013-11-13

**Authors:** Iwao Kukimoto, Tomohiko Maehama, Tsuyoshi Sekizuka, Yumiko Ogasawara, Kazunari Kondo, Rika Kusumoto-Matsuo, Seiichiro Mori, Yoshiyuki Ishii, Takamasa Takeuchi, Toshiyuki Yamaji, Fumihiko Takeuchi, Kentaro Hanada, Makoto Kuroda

**Affiliations:** 1 Pathogen Genomics Center, National Institute of Infectious Diseases, Tokyo, Japan; 2 Department of Biochemistry and Cell Biology, National Institute of Infectious Diseases, Tokyo, Japan; 3 NTT Medical Center Tokyo, Tokyo, Japan; Albert Einstein College of Medicine, United States of America

## Abstract

Viral genetic diversity within infected cells or tissues, called viral quasispecies, has been mostly studied for RNA viruses, but has also been described among DNA viruses, including human papillomavirus type 16 (HPV16) present in cervical precancerous lesions. However, the extent of HPV genetic variation in cervical specimens, and its involvement in HPV-induced carcinogenesis, remains unclear. Here, we employ deep sequencing to comprehensively analyze genetic variation in the HPV16 genome isolated from individual clinical specimens. Through overlapping full-circle PCR, approximately 8-kb DNA fragments covering the whole HPV16 genome were amplified from HPV16-positive cervical exfoliated cells collected from patients with either low-grade squamous intraepithelial lesion (LSIL) or invasive cervical cancer (ICC). Deep sequencing of the amplified HPV16 DNA enabled *de novo* assembly of the full-length HPV16 genome sequence for each of 7 specimens (5 LSIL and 2 ICC samples). Subsequent alignment of read sequences to the assembled HPV16 sequence revealed that 2 LSILs and 1 ICC contained nucleotide variations within E6, E1 and the non-coding region between E5 and L2 with mutation frequencies of 0.60% to 5.42%. In transient replication assays, a novel E1 mutant found in ICC, E1 Q381E, showed reduced ability to support HPV16 origin-dependent replication. In addition, partially deleted E2 genes were detected in 1 LSIL sample in a mixed state with the intact E2 gene. Thus, the methods used in this study provide a fundamental framework for investigating the influence of HPV somatic genetic variation on cervical carcinogenesis.

## Introduction

Human papillomaviruses (HPVs) are small DNA viruses having a circular double-stranded DNA genome of approximately 8-kb, some of which can induce benign and malignant hyper-proliferative lesions in the skin or mucosa [1]. A subset of mucosal HPVs, referred to as high-risk HPVs, are causally related to the development of cervical cancer, among which HPV type 16 (HPV16) accounts for at least 50% of cervical cancer cases worldwide [2]. Mucosal HPVs infect basal cells in the cervix and establish their genomes as extrachromosomal episomes. When infected cells migrate from the basal layer and begin the process of differentiation, viral genome amplification is initiated, followed by the induction of viral capsid expression and the assembly of infectious virions in the upper layers of epithelia [3]. This differentiation-dependent propagation of HPVs can manifest clinically as low-grade squamous intraepithelial lesion (LSIL). In contrast to the episomal state in their normal life-cycle, DNAs of high-risk HPVs are frequently found integrated into the host genome in invasive cervical cancer (ICC) [4,5]. Viral DNA integration leads to enhanced expression of two viral transforming genes, E6 and E7, which play critical roles in carcinogenesis [6,7]. 

In recent years next generation sequencing technologies have been increasingly incorporated into viral genomic research with the aim of comprehensively defining viral genomic sequences in clinical samples [8]. HPV genomic research is no exception; the application of ultra high-throughput sequencing has been applied in the identification of known and unknown HPV genotypes in both cervical and cutaneous lesions [9-11]. In these studies, amplification of full-length HPV genome sequences from clinical specimens has been performed using unbiased amplification techniques such as rolling circle amplification or multiple displacement amplification with phi29 DNA polymerase, followed by deep sequencing analyses or next generation sequencing (NGS) [10,11]. 

In addition, the genetic diversity of viral quasispecies in infected cells or tissues, which is a typical characteristic of RNA viruses [12,13], has been extensively examined by employing deep sequencing techniques. RNA viral populations consist of ‘mutant clouds’, rather than genomes with the same nucleotide sequence, due to the fact that they utilize low-fidelity viral RNA polymerases for their genome replication. Viral quasispecies play major roles in many of the biological activities of RNA viruses. For example, their capacity to change cell tropism or host range or to overcome internal and external selective constraints, such as immune responses and antiviral agents, depends on the presence of variants in mutant clouds. Viral genomic diversity in individual clinical specimens has also been described among DNA viruses, including herpes simplex virus type 1 [14] and Epstein-Barr virus (EBV) [15]. Intriguingly, the detection of hypermutated HPV1a and HPV16 genome sequences in plantar warts and precancerous cervical biopsies, respectively, has also been reported [16]. However, the mutational landscape in the full-length HPV genome present in a clinical specimen and its physiological significance in viral carcinogenesis have not yet been investigated in a comprehensive manner. 

In this study, we aimed to utilize NGS to analyze genetic variation in the entire HPV genome isolated from varying individual clinical samples. To this end, we have used a high-fidelity long-range PCR enzyme to amplify full-length HPV16 genomes from cultured cells and clinical samples. We report that PCR primers targeting a short region of the HPV16 genome yielded amplicons covering the entire viral genome sequence in a single reaction. By analyzing the amplified HPV16 DNA on the Illumina next generation sequencer, we have established a series of procedures for reconstructing the full-length HPV16 genome sequence and extracting genetic variations, such as nucleotide substitutions and insertions/deletions, from millions of short read sequences. This work could form the basis for a comprehensive analysis of HPV genetic variation present in individual clinical specimens. 

## Materials and Methods

### Ethics Statement

Human cervical exfoliated cells were collected from patients at NTT Medical Center Tokyo with written informed consent. The study adhered to the declaration of Helsinki and was approved by the Ethics Committee at NTT Medical Center Tokyo. 

### Cell culture

W12 cell clones 20850 (5 to 50 copies of HPV16 episomes per cell) and 20863 (500 to 1,000 copies of HPV16 episomes per cell) were cultured in the presence of mitomycin C-treated 3T3m feeder cells as described previously [17,18]. CaSki and human embryonic kidney 293 (HEK293) cells were cultured at 37°C with 5% CO_2_ in DMEM supplemented with 10% fetal bovine serum, 50 U/mL of penicillin, and 50 µg/mL of streptomycin.

### Extraction of episomal and total DNA

To extract episomal HPV DNA from W12 cells, a modified Hirt method was used [19]. Briefly, subconfluent cells on a 6-cm dish were digested with 500 μL of lysis buffer consisting of 25 mM Tris-HCl (pH 7.5), 5 mM EDTA, 0.6% SDS, and 50 μg/mL of RNase A at room temperature for 5 min. After the addition of 350 μL of precipitation buffer consisting of 3 M CsCl, 1 M potassium acetate, and 0.67 M acetic acid, the solution was incubated on ice for 15 min, and precipitated by centrifugation (14,000 x g, 15 min, 4°C). Cleared supernatant was applied onto a Miniprep column (Favorgen, Ping-Tung, Taiwan) and passed through the column by centrifugation (14,000 x g, 1 min). The column was then washed with 750 μL of wash buffer consisting of 10 mM Tris-HCl (pH 7.5), 80 mM potassium acetate, 40 μM EDTA, and 60% ethanol. DNA was eluted with 60 μL of elution buffer consisting of 10 mM Tris-HCl (pH 7.5) and 0.1 mM EDTA. The resultant DNA fraction mainly contains extrachromosomal DNAs. 

Total DNA was isolated from cervical exfoliated cells using the QIAamp DNA blood kit (QIAGEN, Hilden, Germany). The DNA samples were genotyped by PGMY-reverse blot hybridization assay [20,21], and HPV16-positive samples without other detectable HPVs from LSIL and ICC specimens were used for subsequent PCR. Total DNA was extracted from CaSki cells as described above for clinical samples. The concentrations of extracted DNAs were about 80 ng/μL from the cultured cells and 2 to 4 ng/μL from clinical samples.

### Full-circle PCR

PCR was performed in 10 μL consisting of 1× PrimeSTAR^®^ GXL Buffer (Takara, Ohtsu, Japan), 200 μM dNTP mixture, 0.25 μM forward primer, 0.25 μM reverse primer, 0.25 U of PrimeSTAR^®^ GXL DNA polymerase (Takara), and 2 to 4 ng of template DNA. Primers used are as follows: 1742F, 5’-tgc tgt cta aac tat tat gtg tgt ctc-3’; 1873R, 5’-gcg tgt ctc cat aca ctt ca-3’; 1869F, 5’-cac gcc aga atg gat aca aa-3’; 1746R, 5’-cag caa ttt ttc aat tgt ttc tc-3’. Positions of 5’-end nucleotide of primers in the reference HPV16 genome (HPV16REF in Papillomavirus *Episteme*, http://pave.niaid.nih.gov) are used as primer names with either forward (F) or reverse (R) orientation. The plasmid containing the full-length HPV16 genome, HPV16/pUC19 [22], was used as a template for positive PCR. The PCR protocol was 98°C for 30 s, followed by 30 cycles of 98°C for 10 s, 60°C for 15 s, 68°C for 2 min. The PCR products were separated on a 0.7% agarose gel in 1 x TAE buffer and visualized with ethidium bromide. 

### Deep sequencing

Approximately 8-kb amplicons generated by full-circle PCR with the primer-pair 1742F/1873R were separated on a 0.7% agarose gel and purified using the Wizard gel purification kit (Promega, Madison, WI). To prepare DNA libraries compatible with Illumina NGS, the purified DNA (16 ng) was fragmented and adaptor-ligated using the Nextra DNA sample prep kit (Epicentre, Madison, WI), followed by DNA purification with the QIAquick PCR purification kit (QIAGEN). The resultant DNA libraries were amplified using a minimal PCR amplification step with Phusion High-Fidelity DNA polymerase (New England Biolab, Ipswich, MA) as follows: 72°C for 3 min, 95°C for 30 s, followed by 13 cycles of 95°C for 10 s, 62°C for 30 s, 72°C for 3 min. Adaptor-ligated DNAs in the range of 500 to 700 bp were then size-selected by 1% agarose gel electrophoresis and purified using the Wizard kit. Five to 8 libraries from different clinical samples were multiplexed with adaptors containing different index sequences and simultaneously subjected to deep sequencing. Cluster generation and sequencing were performed for 151 cycles on a MiSeq sequencer (Illumina, San Diego, CA) with the MiSeq Reagent kit (300 cycle) (Illumina) according to the manufacturer’s instructions. Fluorescent images were analyzed using the Illumina MCS1.1/RTA1.13.56 base-calling pipeline to obtain FASTQ-formatted sequence data. To construct contiguous DNA sequences (contigs), total sequences of paired-end 151-mer reads were assembled using ABySS-pe v1.3.3 [23] with the following parameters: k80, n1200, c1000, t10, e10, q20, and s160; or k80, n120, c100, t10, e10, q20, and s160. The assembly generated 2 to 20 contigs, followed by further assembly of these sequences into the complete circular sequence by Phrap [24]. 

The fidelity of *de novo* assembly was examined by alignment of paired-end reads to the assembled sequence using Burrows-Wheeler Aligner (BWA) v0.6.1 [25]. Prior to read mapping, read sequences of Phred quality score >20 were selected by PoPoolation v1.2.2 [26], and trimmed for 20 bases at 5’-terminus and 51 bases at 3’-terminus because 5’ and 3’-terminus of reads generate relatively low fidelity sequences. The processed 80-base sequences were mapped to the assembled sequence as a paired-end mode by BWA. The mapping results were visualized by Integrative Genomics Viewer (IGV) v2.0.34 [27] or Tablet v1.12.12.05 [28]. The numbers of paired-end reads obtained from each sample and used for *de novo* assembly are described in Table 1, and the average depth of the HPV16 genome ranged from x 7,624 for ICC sample 6 to x 48,221 for W12 sample. Nucleotide mismatches compared to the assembled reference genome and positions of SNPs in each sample were identified by using SAMtools v0.1.18 [29] with in-house Perl scripts (available upon request) or VarScan v2.3.2 [30]. Based on a quality score confidence threshold of Phred quality score > 30 (error probability < 0.001) that was used to extract variation positions in the read sequences, we defined a position as homogeneous if the mutation frequency is < 0.5% and a position to be heterogeneous if the mutation frequency is > 0.5%. The presence of nucleotide substitutions and insertions/deletions was finally verified by visual inspection of mismatched read sequences using IGV and Tablet.

**Table 1:** *De novo* assembly of complete HPV16 genome sequences from short-read sequence data.

Sample	Cytology	Read number	Length (bp)	Variant lineage*	Average depth (per nucleotide)	DDBJ accession
W12	LSIL	2,539,664	7,904	EUR (A1)	48,221	
#1	LSIL	2,057,386	7,903	EUR (A3)	39,064	AB818687
#2	LSIL	1,739,070	7,906	EUR (A3)	33,020	AB818688
#3	LSIL	3,577,966	7,907	NA (D1)	36,233	AB818689
#4	LSIL	921,386	7,903	AFR2 (C)	17,495	AB818690
#5	LSIL	1,913,428	7,906	As (A4)	19,376	AB818691
#6	ICC	401,546	7,905	As (A4)	7,624	AB818692
#7	ICC	1,349,444	7,905	As (A4)	25,622	AB818693
CaSki	ICC	1,394,918	7,905	EUR (A1)	26,486	

* Sublineage designations based on recent classification by Burk et al. [51] are in parenthesis.

### Transient HPV16 replication assay

An HPV16 origin-positive plasmid was constructed by insertion of a *Pst*I fragment of the HPV16 genome (from 7,005 to 7,906 and 1 to 880) into pGL4.50 (Promega). Expression plasmids for N-terminal FLAG-tagged HPV16 E1 and N-terminal FLAG-tagged HPV16 E2 were constructed by cloning of the codon-optimized cDNA of E1 and E2 into pCMV-β (Clontech, Palo Alto, CA). Expression plasmids for E1 mutants were generated by using the QuickChange Lightning Multi Site-Directed Mutagenesis Kit (Agilent Technologies, La Jolla, CA). Transient replication assays were performed as described by Fradet-Turcotte et al. [31] with some modifications. Briefly, HEK293 cells were plated 20 h before transfection in a 24-well plate at a density of 32,000 cells/well, and transfected with 2 ng of the origin-positive plasmid and 2 ng of pGL4.75 (Promega), together with 20 ng of the E2 expression plasmid and the indicated amounts of E1 expression plasmid using the FuGENE6 reagent (Promega). The total quantity of transfected plasmid DNA was adjusted to 100 ng with the empty plasmid p3xFLAG-CMV10 (Sigma-Aldrich, St. Louis, MO) as carrier DNA. At 72 h after transfection, firefly and *Renilla* luciferase activities were measured using the Dual-Glo Luciferase assay system (Promega) on an ARVO MX luminescence counter (PerkinElmer, Waltham, MA), and the level of replication was quantified as the ratio of the two luciferase activities.

### Nucleotide sequence accession number

All assembled HPV genome sequences have been deposited in the DNA Data Bank of Japan (DDBJ) under accession numbers AB818687 to AB818693 for HPV16, AB819272 to AB819274 for HPV52, and AB819275 to AB819279 for HPV58. The sequence reads of the HPV genomes are available from the DDBJ Sequence Read Archive under accession number DRA001083.

## Results

### Full-circle PCR amplifying full-length HPV16 genome sequences

**Figure 1:**
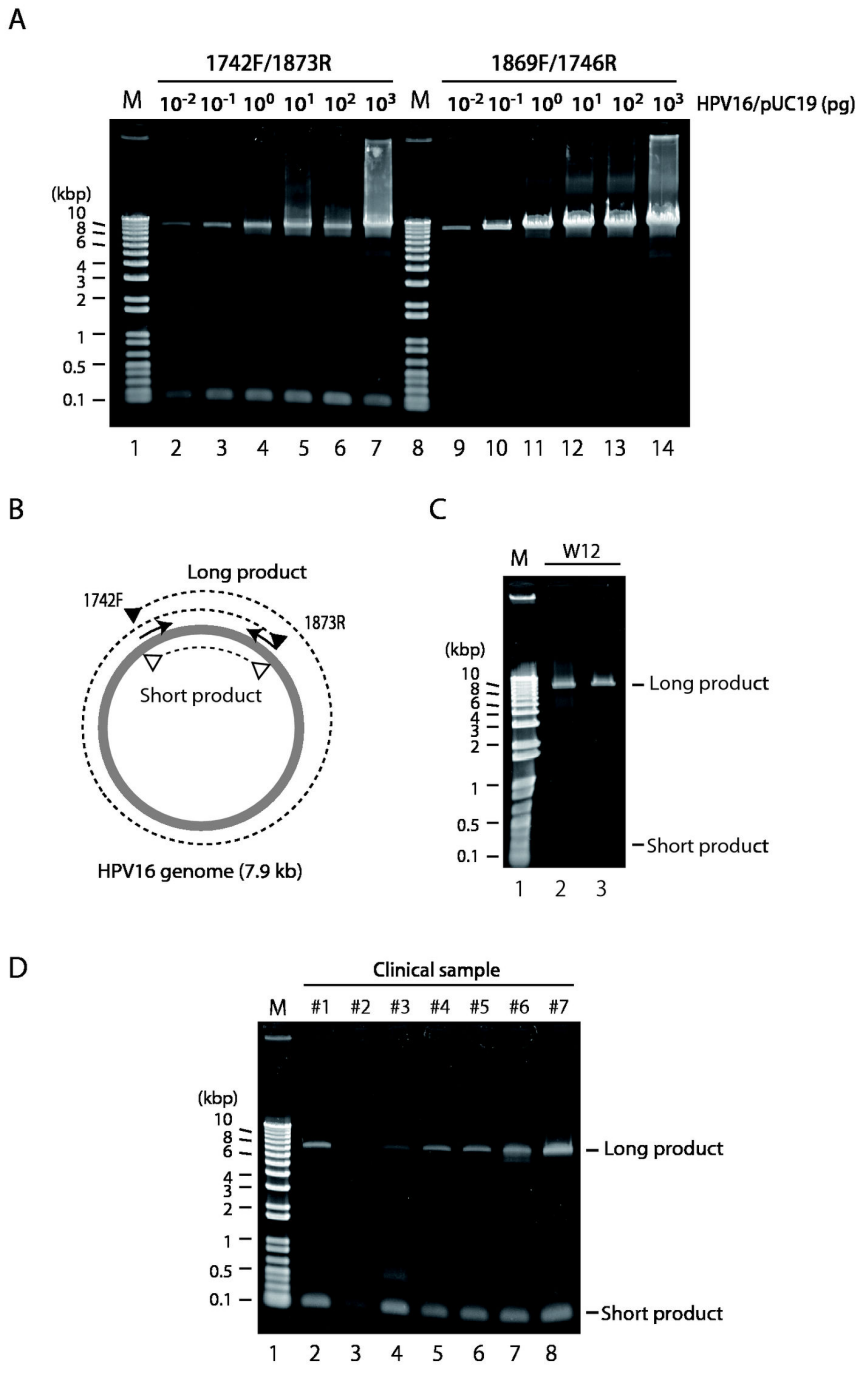
Amplification of full-length HPV16 genomes by full-circle PCR. (A) PCR was performed with PrimeSTAR^®^ GXL DNA polymerase and HPV16-specific primer-pairs as indicated. The amounts of HPV16/pUC19 used for the PCR template are also indicated above. The PCR products were analyzed by agarose gel electrophoresis. M, DNA size markers. (B) Scheme for full-circle PCR. PrimeSTAR^®^ GXL DNA polymerase generates short and long DNA products with primer-pair 1742F/1873R. (C) Full-circle PCR with DNA extracted from W12 cells, clone 20863 (high-copy HPV16 episomes) (lane 2) and clone 20850 (low-copy HPV16 episomes) (lane 3). M, DNA size marker (lanes 1) (D) Full-circle PCR using DNA isolated from 7 clinical specimens: 5 LSIL (lanes 2 to 6), and 2 ICC (lanes 7 and 8). M, DNA size marker (lanes 1).

Using PrimeSTAR^®^ GXL polymerase, which is specifically designed for the fast and accurate amplification of long DNA sequences, and the HPV16-specific primers 1742F and 1873R, we generated two amplicons from HPV16/pUC19 plasmid DNA: a short fragment of ~130 bp and a large fragment consisting of the entire HPV16/pUC19 sequence of approximately 11 kb (with ~130-bp overlap) (Figure 1A, lanes 2 to 7, and see Figure 1B). This PCR reaction is slightly less efficient at creating the large amplicon than the same reaction using the non-overlapping primers 1869F and 1746R, which create only one amplicon (Figure 1A, lanes 9 to 14). The reactions with PrimeSTAR^®^ GXL polymerase were both specific (only 1 or 2 PCR amplicons, depending on the primers used) and sensitive (we successfully amplified as little as 0.01 pg of input template DNA, which corresponds to about 900 copies of the plasmid). The introduction of cellular DNA did not inhibit this reaction, even at the sub-picogram level of template DNA (Figure S1). 

Using the primer-pair 1742F/1873R and PrimeSTAR^®^ GXL polymerase, we also amplified two DNA fragments from extrachromosomal HPV16 DNA isolated from W12 cells that were originally isolated from an LSIL biopsy (Figure 1C). The short and long amplicons matched the predicted sizes of 132 bp and ~8 kb, respectively (Figure 1B), and the long amplicon covered the complete HPV16 genome, as confirmed by Sanger sequencing (results not shown). Hence we refer to this overlapping PCR hereafter as full-circle PCR. To further test the utility of full-circle PCR with PrimeSTAR^®^ GXL polymerase, DNA prepared from CaSki cells, known to contain 60 to 600 copies of integrated tandem repeated HPV16 genomes [32,33], was used as the PCR template. As expected, a mixture of 8 and 6.5-kb amplicons were obtained (Figure S2). 

Importantly, full-circle PCR was able to generate approximately 8-kb amplicons using DNA isolated from HPV16-positive cervical exfoliated cells in patients with either LSIL (Figure 1D, lanes 2 to 6) or ICC (lanes 7 and 8). This observation prompted us to use these long PCR products for genetic variation analyses of full-length HPV16 genomes derived from clinical samples.

### *De novo* assembly of full-length HPV16 genome sequences by deep sequencing

In order to comprehensively analyze HPV genetic variation through deep sequencing, we sought a methodology capable of determining the full-length reference sequence of the HPV16 genome from high-throughput short-read sequence data. First, to develop the system, the 8-kb DNA amplified from W12 DNA by full-circle PCR was fragmented and adaptor-ligated to yield a DNA library compatible with Illumina next generation sequencers. Deep sequencing of the resultant library generated sequence data from more than a million paired-end reads. *De novo* assembly of those paired-end reads by ABySS-pe and Phrap generated one contiguous sequence of 7,904 bp length, which was a perfect match for the full-length W12 HPV16 genome sequence in GenBank (accession No. AF125673). Next, the long PCR products from CaSki DNA were subjected to deep sequencing analysis. The *de novo* assembly procedure once again resulted in construction of one contiguous sequence of 7,905-bp length, identical to the full-length CaSki HPV16 genome sequence in GenBank (accession No. U89348). Mapping of the total read sequences to the CaSki reference sequence demonstrated the presence of two large deletions; from nucleotide positions 1 to 470 and 6,907 to 7,905 (Figure S3), which perfectly matched a deleted region previously reported in CaSki cells [33]. Thus, it is likely that the 6.5-kb DNA products obtained from CaSki DNA contain this 1,469-bp deletion. Overall, these results demonstrate the accuracy of long PCR, deep sequencing and bioinformatics to obtain full-length HPV16 genome sequences. 

To determine the complete genome sequence of HPV16 contained in clinical specimens, we analyzed the 8-kb full-circle PCR products from 7 clinical samples by deep sequencing and assembled the complete viral genome sequence as described above. Table 1 presents the results of this analysis and demonstrates that *de novo* assembly yielded one contiguous sequence for each sample. The length of each sequence ranged from 7,903 to 7,907 bp due to the differences in the length of the non-coding region between E5 and L2 (Figure S4). 

The HPV16 genome is classified into 4 major variant-lineages based on a unique combination of single nucleotide polymorphisms (SNPs): European-Asian, including the sublineages European (EUR) and Asian (As), African 1 (AFR1), African 2 (AFR2), and North-American/Asian-American (NA/AA) [34]. As presented in Table 1, SNP analysis of the assembled HPV16 genomes assigned the following variants to each clinical sample: 2 LSIL samples, EUR; 1 LSIL sample, NA; 1 LSIL sample, AFR2; and 1 LSIL and 2 ICC samples, As. Furthermore, phylogenetic tree analysis with the assembled complete genome sequences of HPV16 confirmed the assignments in variant-lineages (Figure S5). These results indicate that one HPV16 variant predominates in each clinical specimen. 

### Analysis of nucleotide variations in HPV16 genomes

**Figure 2:**
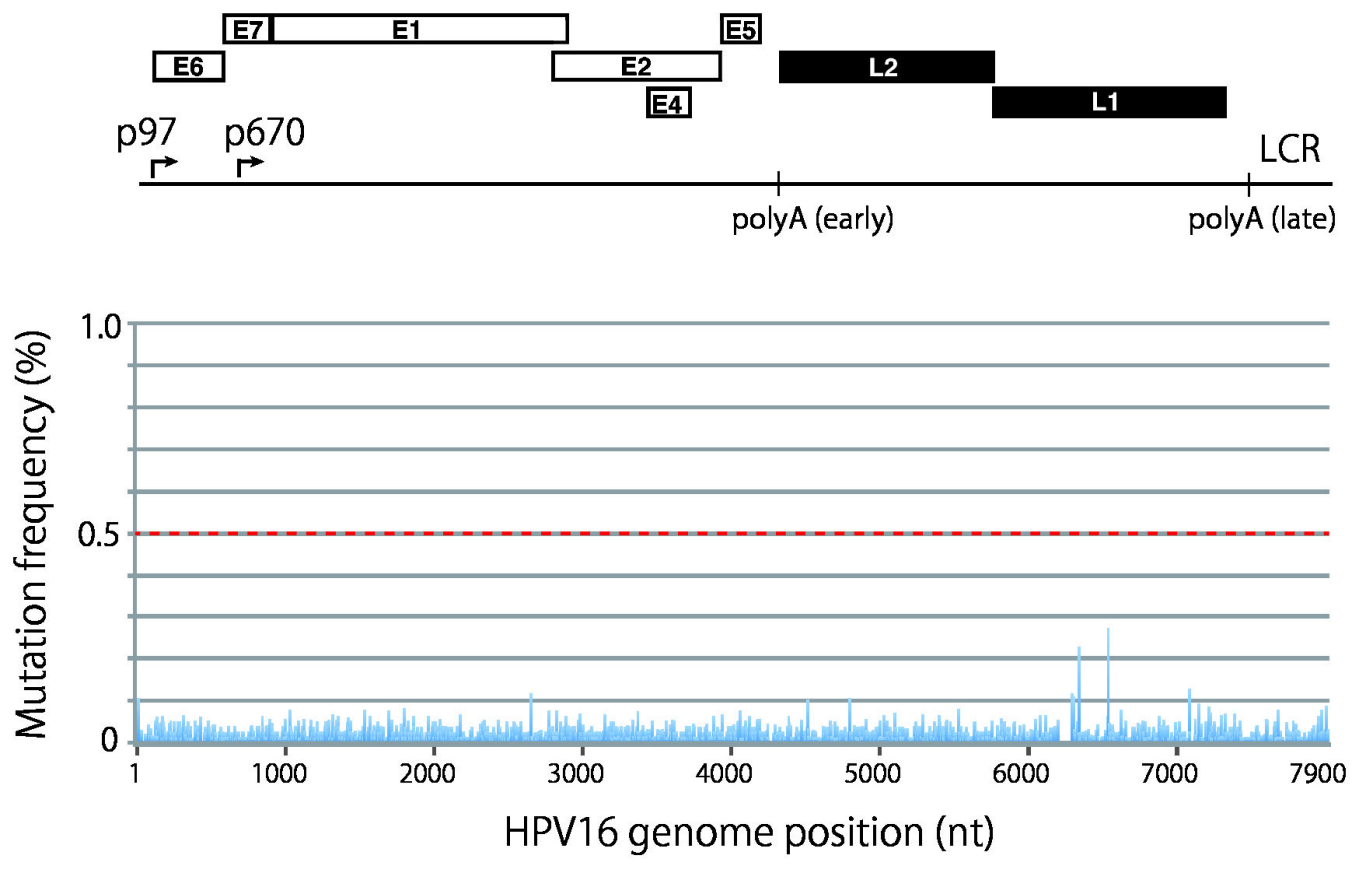
Mutation frequency profile of full-length HPV16 genomes prepared from a plasmid. The read sequences obtained with full-length HPV16 genomes prepared from a cloned plasmid were aligned to the reference HPV16 sequence, and mutation/error frequencies at each nucleotide position relative to the total coverage (number of reads that encompass each nucleotide, up to the maximum x 8,000) are presented in the landscape of the full-length HPV16 genome. A threshold line for a reliable mutation frequency (0.5%) is indicated with the red dotted line. The genome organization of HPV16 is indicated above: p97, the early promoter; p670, the late promoter; polyA(early) and polyA(late), the early and late polyadenylation signals, respectively.

 Before analyzing the sequence variation present in clinical samples, we first validated the efficacy and error rates of deep sequencing on our platform. For this purpose, we used a linearized full-length HPV16 DNA that was excised from HPV16/pUC19 by *Bam*HI digestion. The read sequences obtained were aligned to the prototype HPV16 sequence (GenBank accession No. K02718), and the number of reads containing nucleotide mismatches was determined at each position of the viral genome. This analysis enabled us to calculate mutation frequencies at each nucleotide position (the average depth of reads count per nucleotide position was 7,035). As shown in Figure 2, an overall profile of the mutation/error frequency demonstrated an error rate below 0.3% distributed throughout the whole sequence, which reflects intrinsic errors generated in the deep sequencing analysis. The average error rate per nucleotide position throughout the whole sequence was determined to be 0.0070%. Visualization and manual inspection of aligned read sequences revealed that the two minor peaks observed at nt positions 6,242 and 6,434 (see Figure 2) were derived from mis-mapping of reads to ends of the linear HPV16 DNA (*Bam*HI site, at nt 6,154). 

Our deep sequencing analysis of HPV16 DNA amplified by PrimeSTAR^®^ GXL polymerase demonstrated that the average error rate was 0.0076% (results not shown), which is comparable to the error rate reported by the manufacturer (0.0062%), indicating high fidelity DNA amplification by this polymerase. Based upon this low error rate and our analytical method’s ability to pick up nucleotide variation with a minimum confidence score threshold of Phred quality score 30 (error probability < 0.001), we decided to use 0.5% as a threshold value of mutation frequency to distinguish real mutations from putative errors. 

**Figure 3:**
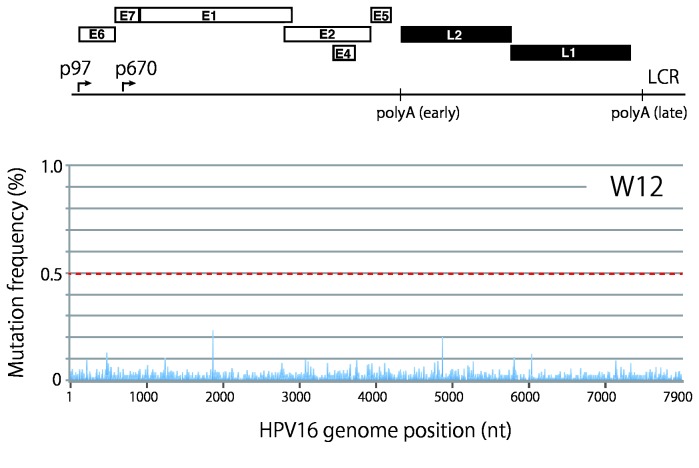
Mutation frequency profile of full-length HPV16 genomes in W12 cells. The read sequences obtained with full-length HPV16 genomes prepared from W12 cells were aligned to the reference HPV16 sequence (AF125673), and mutation/error frequencies at each nucleotide position are presented in the landscape of the full-length HPV16 genome. A threshold line for a reliable mutation frequency (0.5%) is indicated with the red dotted line. The genome organization of HPV16 is indicated above.

We applied this variation analysis method to HPV16 genome sequences obtained from W12 cells. As shown in Figure 3, alignment of the paired-end reads to the W12 reference sequence revealed no major nucleotide substitutions with a frequency >0.5% in the read coverage , suggesting that HPV16 genome sequences are highly homogeneous in W12 cells. 

**Figure 4:**
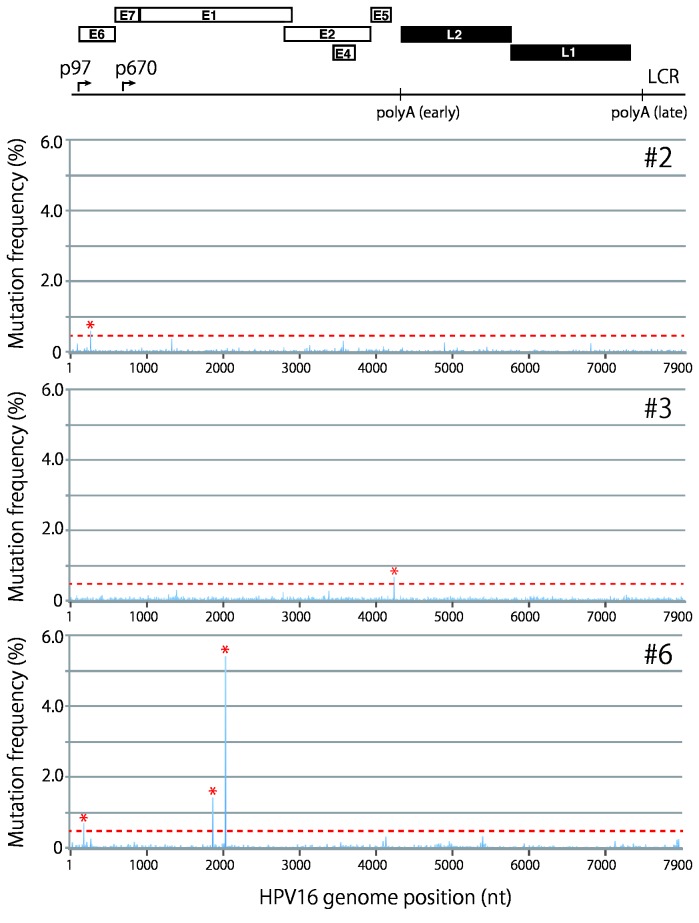
Mutation frequency profile of full-length HPV16 genomes in clinical specimens. The read sequences obtained with full-length HPV16 genomes prepared from clinical specimens (2 LSIL samples, #2 and #3; 1 ICC sample, #6) were aligned to their *de* *novo* assembled complete genome sequences, and mutation/error frequencies at each nucleotide position are presented in the landscape of the full-length HPV16 genome. A threshold line for a reliable mutation frequency (0.5%) is indicated with the red dotted line. Peaks above 0.5% are indicated with red asterisks. The genome organization of HPV16 is indicated above.

 Next we employed this established analysis procedure to examine HPV genomic variation in clinical samples. We report two types of mutation profiles found in 7 clinical samples: a highly homogeneous profile as observed with W12 cells, and a profile with 1 to 3 specific positions of nucleotide substitution. Four samples (#1, #4, #5, and #7) showed an almost homogeneous profile without specific nucleotide substitution (two representative profiles are presented in Figure S6) and 3 samples (#2, #3, and #6) demonstrated a mutation profile with several positions of nucleotide substitution (Figure 4). The nucleotide substitutions were located in the early region of the HPV16 genome, E6 and E1, and the non-coding region between E5 and L2, with frequencies from 0.60 to 5.42% (Table 2). Intriguingly, the nucleotide substitutions observed in the E1 gene of sample 6 cause amino-acid (aa) changes in the E1 protein: methionine at aa position 326 to isoleucine, and glutamine at aa position 381 to glutamic acid. The nucleotide substitution in the E6 gene detected in sample 6 also leads to an aa change in the E6 protein, glutamic acid to aspartic acid at aa position 25.

**Table 2:** Nucleotide and amino acid changes detected in individual clinical specimens.

Sample	Nt position	Nt change	*Frequency (%)	Aa position	Aa change	Gene
#2	256	C to T	0.60	51	no change	E6
#3	4158	A to G	0.70	-	no change	-
#6	178	G to T	0.72	25	Glu to Asp	E6
	1842	G to A	1.44	326	Met to Ile	E1
	2005	C to G	5.42	381	Gln to Glu	E1

* Mutation frequencies were calculated by using SAMtools with in-house Perl scripts.

 We were also able to employ our full-circle PCR and deep sequencing approach to analyze clinical samples that contained HPV52 and HPV58 DNA (Figure S7 and Table S1), suggesting that this method is not specific to the analysis of HPV16 and that it is also applicable to the oncogenic HPV types prevalent in East Asian countries including China and Japan [35-37]. 

### Effect of E1 amino-acid changes on its replication function

**Figure 5:**
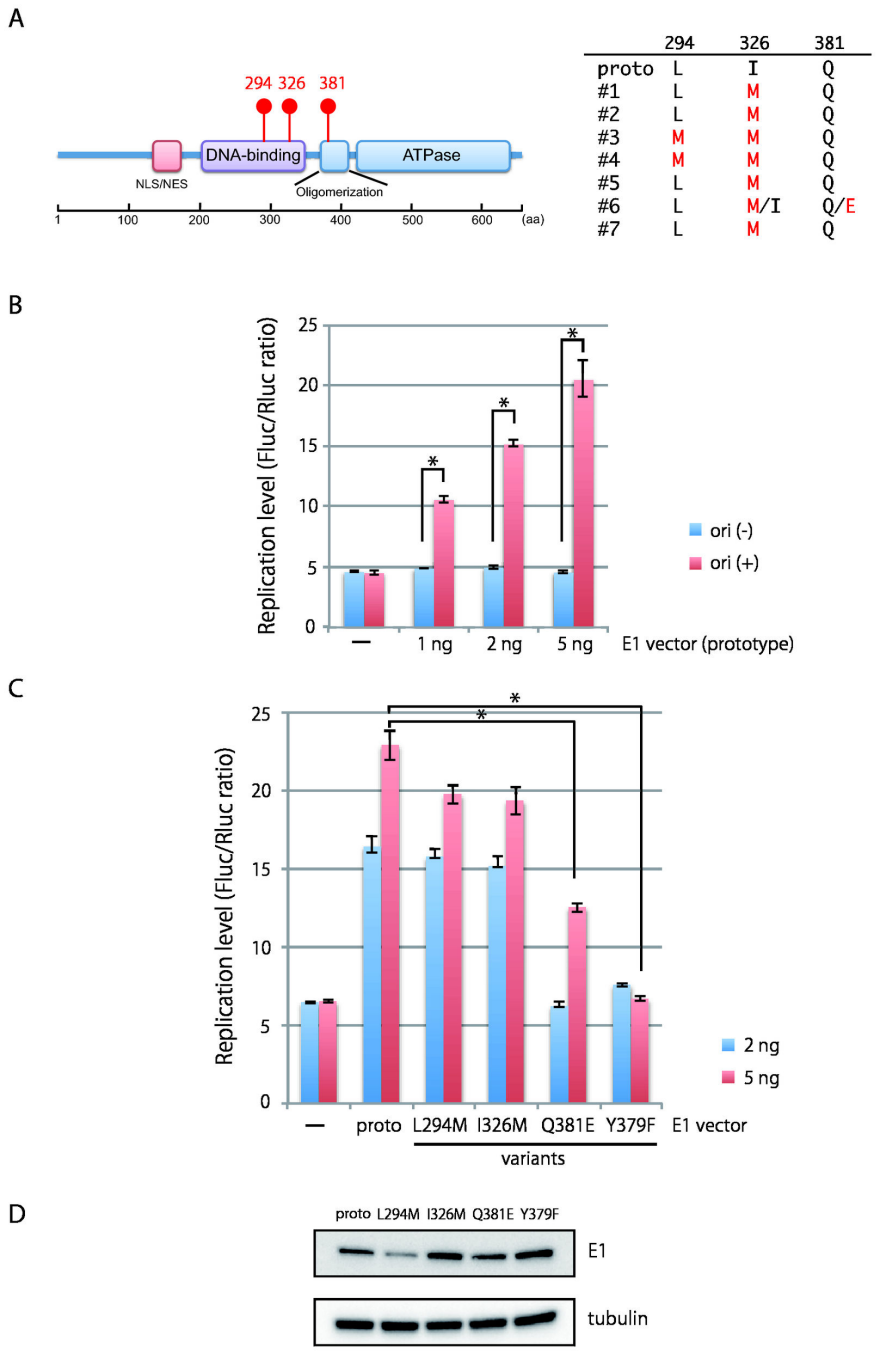
Characterization of E1 variants by transient HPV16 replication assay. (A) Left panel: schematic representation of the HPV16 E1 protein. The prototype E1 protein has leucine at position 294 and isoleucine at position 326, both of which are located in the DNA-binding domain, and glutamine at position 381 located in the oligomerization domain. Right panel: summary of aa variations found in the E1 protein from the clinical samples (#1 to #7). Proto, the E1 protein of European prototype. (B) E1-dependent replication of HPV16 origin-positive plasmid. The HPV16 origin-positive plasmid expressing Firefly luciferase (Fluc) and the origin-deficient plasmid expressing *Renilla* luciferase (Rluc) were transfected into HEK293 cells with increasing amounts of the prototype E1 expression plasmid together with the E2 expression plasmid. At 72 h after transfection, the ratio of the two luciferase activities (Fluc/Rluc) was measured as levels of the origin-dependent replication. The origin-deficient plasmid expressing Firefly luciferase (pGL4.50) was used as a negative control for HPV replication. Statistically significant differences (Welch’s t-test, *p*<0.01) are indicated with *. (C) Replication activity of E1 variants. Increasing amounts of expression plasmids for FLAG-tagged prototype or variant E1 proteins were transfected into HEK293 cells, and the levels of replication were measured at 72 h after transfection. Error bars represent the standard deviation of triplicate transfections. Statistically significant differences (Welch’s t-test, *p*<0.01) are indicated with *. The data are representative of three independent experiments. (D) Western blot analysis of E1 variants. FLAG-tagged prototype or variant E1 proteins expressed in HEK293 cells were detected with anti-FLAG antibody (Sigma-Aldrich) and the ECL prime Western blotting detection reagent (GE Healthcare, Buckinghamshire, England). Tubulin was visualized with anti-α/β-tubulin antibody (Cell Signaling, Danvers, MA) as loading control.

 The *de novo* assembled HPV16 genomes from clinical specimens revealed aa polymorphisms of the E1 protein in its DNA-binding domain at aa positions 294 and 326: a replacement of leucine (prototype) at position 294 with methionine (L294M) in 2 LSIL specimens (#3 and #4) and that of isoleucine (prototype) at position 326 with methionine (I326M) in all the specimens (Figure 5A). Further, nucleotide variation analyses showed a novel aa variation at position 381 from glutamine (prototype) to glutamic acid (Q381E) in sample 6. To explore the biological significance of these E1 variations, we mutated the corresponding aa residues in the European prototype E1 protein and tested the mutated E1’s ability to support HPV16 origin-dependent DNA replication. The E1 mutant Y379F (in which tyrosine at 379 is replaced with phenylalanine), which is expected to lose replication activity as reported previously [38], was also included as a representative of a replication-deficient E1 protein. 

In the transient HPV16 replication assay, two reporter plasmids were transfected into HEK293 cells together with expression vectors for FLAG-tagged E1 and E2. Firefly luciferase (Fluc) is expressed from the HPV16 origin-positive plasmid, whereas *Renilla* luciferase (Rluc) is expressed from the origin-deficient plasmid, so the ratio of the two luciferase activities (Fluc/Rluc) in the presence of the E1/E2 expression vectors represents levels of HPV origin-dependent replication. As previously reported [31], the Fluc/Rluc ratio obtained with the origin-positive plasmid increased in a manner dependent on the amount of the E1 expression plasmid (Figure 5B). Under these assay conditions, two E1 variants, L294M and I326M, showed replication activities almost comparable to the parental E1 (Figure 5C). In contrast, Q381E exhibited a reduced activity for supporting HPV16 replication, and Y379F completely lost the replication activity as expected. Western blot analyses revealed that the expression levels of I326M, Q381E, and Y379F were comparable to that of the prototype E1 (Figure 5D), indicating that the reduced replication activity of Q381E is not due to reduced protein level. 

### Detection of deletions in HPV16 genomes

**Figure 6:**
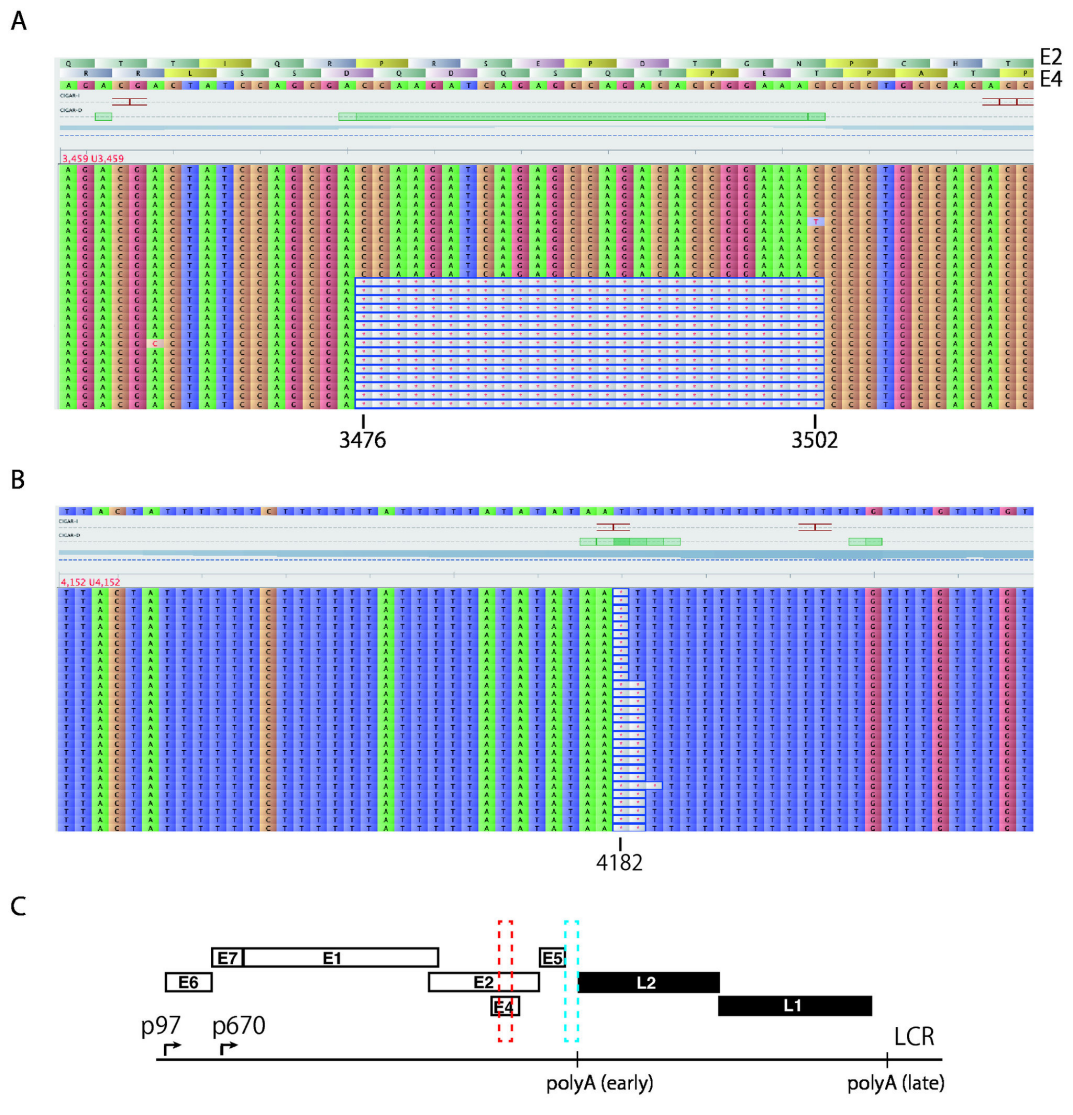
HPV16 genome deletions in a clinical specimen revealed by read mapping. Mapping of paired-end read sequences to *de* *novo* assembled full-length HPV16 genome sequence for LSIL sample 2 is visualized by Tablet. Deletions in the E2/E4 region (A) and in the region between E5 and L2 (B) are shown. (C) The genome organization of HPV16. The deleted regions in E2/E4 and between E5 and L2 are indicated with red and blue dotted-line boxes, respectively.

Because our deep sequencing analysis was able to precisely define the deletion in the HPV16 genome of CaSki cells, we examined HPV16 genome deletions in the 7 clinical samples by read mapping to the assembled reference sequence. Of the 5 LSIL samples, 4 LSIL samples did not show any deletions and 1 LSIL sample (#2) demonstrated the discontinuous distribution of read depth indicative of genome deletions (Figure S3, arrows). Further detailed visualization of the mapped read sequences revealed that 27 bases were deleted from nucleotide positions 3,476 to 3,502 in 78.9% of total read sequences covering this region (Figure 6A). Independent full-circle PCR for sample 2 and subsequent deep sequencing analysis yielded a comparable deletion frequency (86.6%, results not shown), demonstrating the reproducibility of the analysis overall. PCR amplification of the E2 region (Figure S8) and subsequent Sanger sequencing confirmed the presence of the deletion (results not shown). In addition, read sequences lacking 1 to 3 bases in the region between E5 and L2 were also detected in sample 2 in 89.0% of the total read coverage of that region (Figure 6B). Although the read depth in the E5 region was low (Figure S3), no deletion was found in this region. These data suggest that deleted viral genomes are present as a mixed state with intact full-length HPV16 genomes in this clinical specimen. In contrast to HPV16 genomes in CaSki cells, no deletion was detected in HPV16 genome sequences obtained from the 2 ICC samples.

## Discussion

In this study, utilizing a recently developed long PCR enzyme, PrimeSTAR^®^ GXL polymerase, we have succeeded in amplifying full-length HPV16, HPV52, and HPV58 genome sequences, using DNA isolated from clinical specimens, in a single reaction. The use of this highly-processive DNA polymerase is critical, since traditional PCR enzymes such as *Taq* DNA polymerase are unable to amplify such long DNAs. Furthermore, by applying the NGS technology to the amplified HPV DNA, we have developed a methodology capable of analyzing the genetic variation present in individual clinical samples. Although library construction is still laborious, an improved Nextera DNA sample preparation kit (Illumina) facilitates rapid and simple library preparation for up to 96 samples with different index primers, thus allowing each sample to be analyzed in a single NGS run and further reducing the overall cost. 

The *de novo* assembly procedure described in this study provides a simple and reliable methodology to construct a reference sequence of the full-length HPV genome from millions of short-read sequences. Construction of a contiguous reference sequence from short-read sequence data is critical for both determining the complete genome sequence of known or unknown HPVs and examining sequence variations in HPV genomes compared to the reference sequence. In this regard, it is worth noting that the overlapping sequence at both ends of the long PCR products (see Figure 1B) contributes to an accurate and faithful reconstruction of the circular HPV genome. 

By aligning short-read sequences to the assembled reference sequence and extracting nucleotides that do not match the reference sequence, we have presented a comprehensive profile of HPV genetic variation in the landscape of the whole viral genome. Overall, HPV16 genome sequences in individual cervical specimens showed a highly homogeneous profile. Among the 7 clinical samples, 4 samples did not contain nucleotide substitution with a frequency >0.5% anywhere in their genomes, suggesting that HPV16 genome sequences in infected cells of the cervix are highly static. However, the remaining 3 samples demonstrated nucleotide substitutions (1 to 3 positions) with a frequency >0.5% in the total read coverage (Figure 4). The mutations were located in E6, E1 or the non-coding region between E5 and L2, and the levels of the mutation frequency ranged from 0.60 to 5.42%, which is considerably lower than those observed for other viruses, such as HCV and HBV [13]. The low frequency of mutation in the HPV genome likely reflects its strategy for cellular replication that relies completely on host DNA polymerases [39]. Human DNA polymerases have proof-reading activities that enable faithful replication of the host genome, thus also contributing to the accurate replication of HPV genomes. Nevertheless, we cannot completely exclude the possibility of co-infection by highly related HPV16 variants. In particular, since A at nucleotide position 1842 is a diagnostic SNP to assign the EUR sublineage [40], the G to A variation observed at this position in sample 6 (Table 2) may suggest the coexistence of two closely related variants, As and EUR, in a single specimen. However, no read sequences containing other diagnostic SNPs for EUR (C at nucleotide position 3159, G at nucleotide position 3249, and C at nucleotide position 3787) were detected in this sample.

One possible mechanism for the introduction of low-frequency mutations into the HPV16 genome is an impairment of the p53-dependent DNA damage response (DDR) by the E6 protein. To preserve genetic information when DNA is damaged, eukaryotic cells evoke the DDR involving detection of the DNA damage by sensor proteins, transduction of the signal from DNA lesions to inhibit cell cycle progression and recruitment of repair factors [41]. In the course of the DDR, p53 plays important roles in promoting cell survival by activating cell-cycle arrest and DNA repair, or cell death by directing cells to undergo apoptosis thus eliminating extensively damaged cells. To overcome the functions of p53 and create a cellular environment that favors viral propagation, the E6 protein of high-risk HPVs binds to and inactivates p53 via proteasomal degradation [7]. In cervical basal cells retaining HPV genomes, or differentiating cells supporting HPV genome amplification, E6 may facilitate the introduction of mutations into both cellular and HPV genomes through inhibition of the p53-dependent DDR. 

The mutations found in the E1 gene are non-synonymous substitutions and cause aa changes in the E1 protein, a DNA helicase that plays essential roles in the initiation and progression of viral genome replication. While the amino-acid replacements at positions 294 and 326 located in the DNA-binding domain (see Figure 5A), and seen in multiple HPV16 lineages [40], did not exert a major effect on the replication activity of E1, the previously unreported mutation at position 381 found in 1 ICC sample led to reduced HPV replication. Since glutamine at 381 is located in the oligomerization domain of E1 (Figure 5A) and the nearby Y379F mutant completely lost replication activity (Figure 5C), possibly due to a failure to form E1-E1 oligomers [38], we suggest that the Q381E mutation may also affect oligomerization of E1. Although amino-acid polymorphisms in E1 have been previously reported for HPV18 and are thought to be responsible for functional differences between HPV18 E1 intra-type variants [42], our present study suggests the presence of a mixture of E1 variants in a single clinical specimen as previously reported for HPV62 [43]. We have not yet determined whether the HPV16 genomes containing the E1 mutations were episomal or integrated, because full-circle PCR is expected to amplify tandem multiple-copies of the HPV16 genome, that are occasionally found in cervical cancer [4], and as we observed with CaSki cells (Figure S2). Frame-shift mutations in the E1 open-reading frame have also been reported for several HPV genotypes including HPV16 [43], and we suggest that any loss of E1 activity due to E1-disrupted genomes can be compensated by E1 proteins produced from E1-intact genomes. Although the physiological impact of the mixed state of multiple E1 proteins on HPV life cycle is not clear, somehow the attenuated replication phenotype of the E1 protein, as observed with Q381E, may facilitate the oncogenic progression of HPV-infected lesions, since E1 functions are frequently silenced or lost during progression to cervical cancer. 

 The 27-bp deletion found in the E2/E4 region in 1 LSIL specimen causes in-frame deletions in both the E2 and E4 proteins, which may have an impact on their functions. The E2 protein is a site-specific DNA-binding protein required for HPV replication, transcription and genome segregation, and consists of an amino-terminal *trans*-acting domain, a central hinge domain, and a carboxyl-terminal protein dimerization and DNA-binding domain [44]. Because the deletion is located in the E2 hinge domain that plays a role in nuclear localization and nuclear matrix association [45], it may impair these activities. Intriguingly, deletions in the E2 hinge region have previously been reported in about 80% of cervical carcinoma in situ and invasive cervical cancer specimens [46]. Given that the E2 protein may be a transcriptional repressor of the HPV16 promoter responsible for E6/E7 expression, and its functional loss by integration into the host genome may contribute to up-regulation of the E6/E7 expression and development of cervical cancer [1], further studies with larger sample sizes will be needed to assess whether the deletion of the hinge domain has any general consequences for the progression of precancerous lesions to ICC.

Although a previous study demonstrated the presence of A/T-rich hypermutation in the LCR of HPV16 in precancerous lesions [16], we could not detect such hypermutated sequences with a frequency >0.5% in the total coverage at any positions in the viral genome. This discrepancy is likely due to the extremely high sensitivity of differential DNA denaturation-PCR (3D-PCR) utilized in the previous study to detect hypermutated A/T-rich DNA. To denature target DNA, 3D-PCR uses lower melting temperatures than that used in conventional PCR, which favors selective amplification of A/T-rich DNA and results in enrichment of A/T-rich mutated DNA in the final PCR product. Thus 3D-PCR suggests the presence of hypermutation in the HPV genome, but provides no information about the overall frequency of such hypermutation events. In contrast, we have used an unbiased PCR method to recover the full-length HPV16 genome sequence in a single reaction: a method that is more suitable for capturing the overall frequency of mutation throughout the viral genome. Our analysis of mutation frequency suggests that if such A/T-rich hypermutation were present the frequency is below 0.5%, suggesting that it constitutes only a minor fraction of the viral population. The exact frequency and physiological significance of A/T-rich hypermutation requires further investigation.

Lastly, as shown in this study, deep sequencing of the full-length HPV genome allows comprehensive analyses of those genetic variations, including base substitutions and insertions/deletions, which occur during persistent HPV infection and that may have a link to disease progression. Further investigation of full-length HPV genome sequences in individual clinical samples is essential for a better understanding of the genetic and epidemiological basis for HPV-associated carcinogenesis because some E6 and long control region variants of HPV16 are known to possess enhanced oncogenicity [47-49] and HPV16 variant lineages show different distributions in cervical cancer cases by country and region [50]. Although the level of genetic variation in HPV genomes isolated from cervical specimens observed in this study is seemingly low, its influence on carcinogenesis, diagnostics, and clinical management will undoubtedly be an important topic for future HPV research.

## Supporting Information

Table S1 ***Denovo* assembly of complete HPV52/58 genome sequences from short-read sequence data.**
(DOC)

Figure S1 **Sensitivity and specificity of full-circle PCR.** Full-circle PCR was performed with PrimeSTAR^®^ GXL DNA polymerase and the primer-pair 1742F/1873R in the presence (lanes 2 to 6) or absence (lanes 8 to 12) of cellular DNA (10 ng per reaction). The amounts of HPV16/pUC19 used for the PCR template are also indicated. M, DNA size markers.
(PDF)

Figure S2 **Amplification of full-length HPV16 genomes from CaSki cells.** (A) Scheme for PCR amplification from integrated HPV16 genomes in cervical cancer. Tandem multiple-copies of integrated HPV16 DNA can be a template for full-circle PCR as well as episomal HPV16 genomes, while single-copy integrated HPV16 DNA cannot. (B) PCR was performed with primer-pair 1742F/1873R and increasing amounts of total DNA extracted from CaSki cells (lanes 2 and 3). M, DNA size marker (lane 1).
(PDF)

Figure S3 **Read-depth profile obtained by deep sequencing of long PCR products.** (A) Paired-end read sequences were aligned using BWA to each *de* *novo* assembled complete HPV16 genome sequence, and resultant read-depth profiles for CaSki and LSIL sample 2 are shown. Arrows indicate positions of the discontinuous distribution of read depth. The maximum read-depths were 30,173 for CaSki and 23,901 for sample 2. (B) Scheme for the genomic organization of HPV16.
(PDF)

Figure S4 **Alignment of *denovo* assembled HPV16 genome sequences.** Eight *de* *novo* assembled complete HPV16 genome sequences (samples 1 to 7, and W12) were aligned against each other by MAFFT. The sequence alignment around the non-coding region between E5 and L2 is presented. The stop codon of E5 is indicated with dotted-line box. Numbering of nucleotide positions is based on the sequence of #1.
(PDF)

Figure S5 **Phylogenetic analysis of *denovo* assembled complete HPV16** **genome sequences**. Sixty-two complete HPV16 genome sequences previously reported (Smith et al., PLoS One: 6, e21375, 2011) and 7 complete HPV16 genome sequences *de* *novo* assembled in this study were aligned against each other by mafft. Maximum likelihood phylogenetic tree was constructed using the aligned sequences with 1,000-fold bootstrapping. The scale indicates a branch length of 0.01 that shows 1% difference between the nucleotide sequences at the beginning and end of the branch. The number at each branch node represents the bootstrapping value. Vertical bars indicate major clades of HPV16 variants: AFR1, AFR2, NA, AA, As, and EUR.
(PDF)

Figure S6 **Mutation frequency profile of full-length HPV16 genomes in clinical specimens.** The read sequences obtained with full-length HPV16 genomes prepared from clinical specimens (#1, LSIL; #7, ICC) were aligned to their *de* *novo* assembled complete genome sequences, and mutation/error frequencies at each nucleotide position are presented in the landscape of the full-length HPV16 genome. A threshold line for a reliable mutation frequency (0.5%) is indicated with the red dotted line. The genome organization of HPV16 is indicated above.
(PDF)

Figure S7 **Amplification of full-length genomes of HPV52 and HPV58 from clinical specimens.** Full-circle PCR was performed with HPV52 or HPV58-specific primers and DNA isolated from 8 HPV52-positive LSIL (lanes 1 to 8) and 8 HPV58-positive LSIL (lanes 10 to 17) specimens. Primers are as follows: HPV52-F, 5’-ACC AGA AAC ACA TAT GGT AAT AGA ACC-3’; HPV52-R, 5’-GTA ATA CTG TTT GTT GTT CTA TCC ATT C-3’; HPV58-F, 5’-TAC TAT CAA TTC CTG AAA CAT GTA TGA-3’; HPV58-R, 5’-AAT CTA TCT ATC CAT TCT GGT GTT G-3’. M, DNA size marker (lane 9).
(PDF)

Figure S8 **Amplification of deleted E2 sequence from a clinical specimen.** Conventional PCR using AmpliTaq Gold was performed with HPV16-specific primers in the E2 gene and DNA extracted from LSIL specimen 2 (lane 2) and HPV16/pUC19 (lane 3). M, DNA size marker (lane 1).
(PDF)
