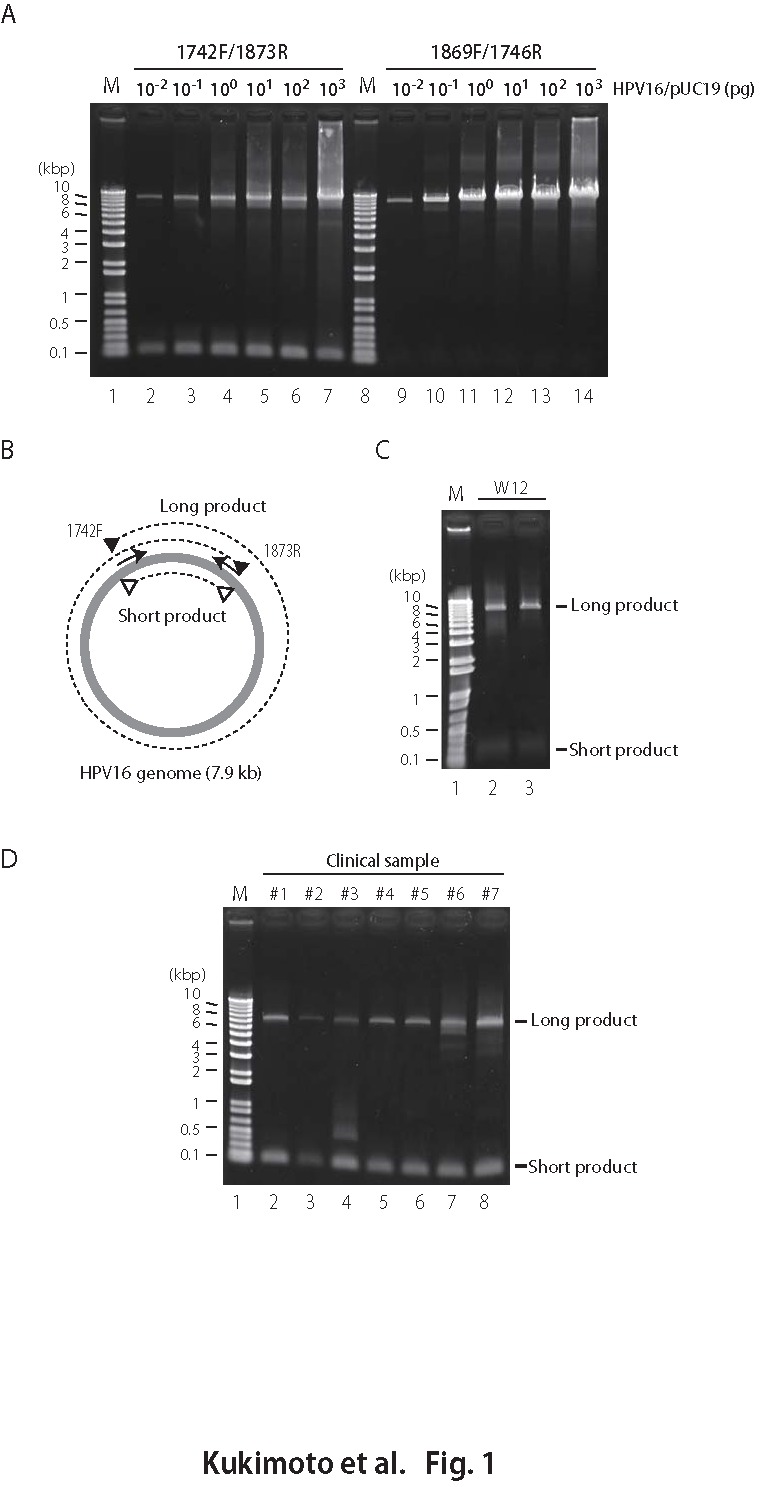# Correction: Genetic Variation of Human Papillomavirus Type 16 in Individual Clinical Specimens Revealed by Deep Sequencing

**DOI:** 10.1371/annotation/524fa93c-e77c-4c3d-bf6e-763a5b057409

**Published:** 2014-01-16

**Authors:** Iwao Kukimoto, Tomohiko Maehama, Tsuyoshi Sekizuka, Yumiko Ogasawara, Kazunari Kondo, Rika Kusumoto-Matsuo, Seiichiro Mori, Yoshiyuki Ishii, Takamasa Takeuchi, Toshiyuki Yamaji, Fumihiko Takeuchi, Kentaro Hanada, Makoto Kuroda

An error was introduced during the production process. The publisher apologizes for this error. In Figure 1D, the long product band in lane 2 is not visible. The correct version of Figure 1 can be viewed here: 

**Figure:**