# Supplementary material for: Genetic Variation of Human Papillomavirus Type 16 in Individual Clinical Specimens Revealed by Deep Sequencing
Source: PLoS One. 2013 Nov 13;8(11):e80583. doi: 10.1371/journal.pone.0080583 (PMC3827439; doi:10.1371/journal.pone.0080583)
Supplement: Table S1 — Denovo assembly of complete HPV52/58 genome sequences from short-read sequence data. (DOC) [file pone.0080583.s001.doc]

**Table S1. *De novo* assembly of complete HPV52/58 genome sequences from short-read sequence data.**

| Sample | Cytology | HPV52/58 | Read number | Length (bp) | Variant sublineage | DDBJ accession |
| --- | --- | --- | --- | --- | --- | --- |
| #8 | LSIL | HPV52 | 6,014,244 | 7,960 | B2 | AB819272 |
| #9 | LSIL | HPV 52 | 2,593,108 | 7,960 | B2 | AB819273 |
| #10 | LSIL | HPV 52 | 8,143,518 | 7,960 | B2 | AB819274 |
| #11 | LSIL | HPV 58 | 2,553,782 | 7,824 | A2 | AB819275 |
| #12 | LSIL | HPV 58 | 8,270,782 | 7,824 | A2 | AB819276 |
| #13 | LSIL | HPV 58 | 2,003,584 | 7,824 | A2 | AB819277 |
| #14 | LSIL | HPV 58 | 1,141,218 | 7,824 | A2 | AB819278 |
| #15 | LSIL | HPV 58 | 1,745,566 | 7,836 | A3 | AB819279 |
