# Supplementary figures and images for: Genetic Variation of Human Papillomavirus Type 16 in Individual Clinical Specimens Revealed by Deep Sequencing
Source: PLoS One. 2013 Nov 13;8(11):e80583. doi: 10.1371/journal.pone.0080583 (PMC3827439; doi:10.1371/journal.pone.0080583)

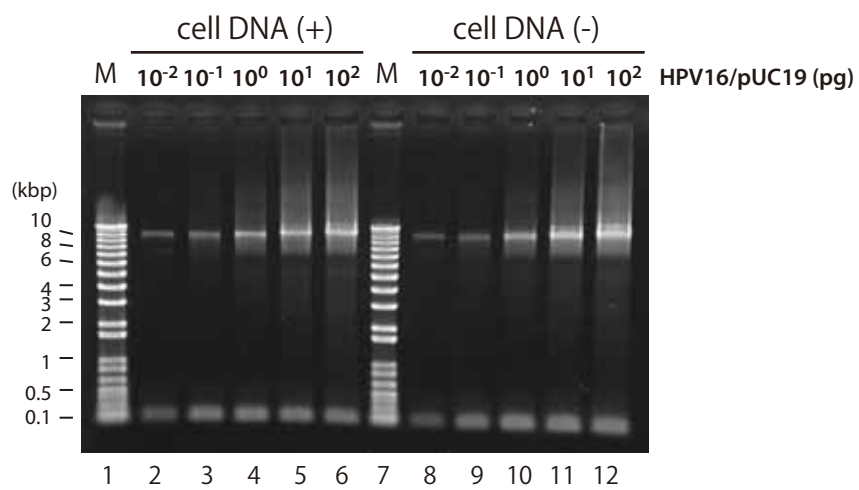

Kukimoto et al. Fig. S1

Supplement: Figure S1 — Sensitivity and specificity of full-circle PCR. Full-circle PCR was performed with PrimeSTAR® GXL DNA polymerase and the primer-pair 1742F/1873R in the presence (lanes 2 to 6) or absence (lanes 8 to 12) of cellular DNA (10 ng per reaction). The amounts of HPV16/pUC19 used for the PCR template are also indicated. M, DNA size markers. (PDF) [file pone.0080583.s002.pdf]

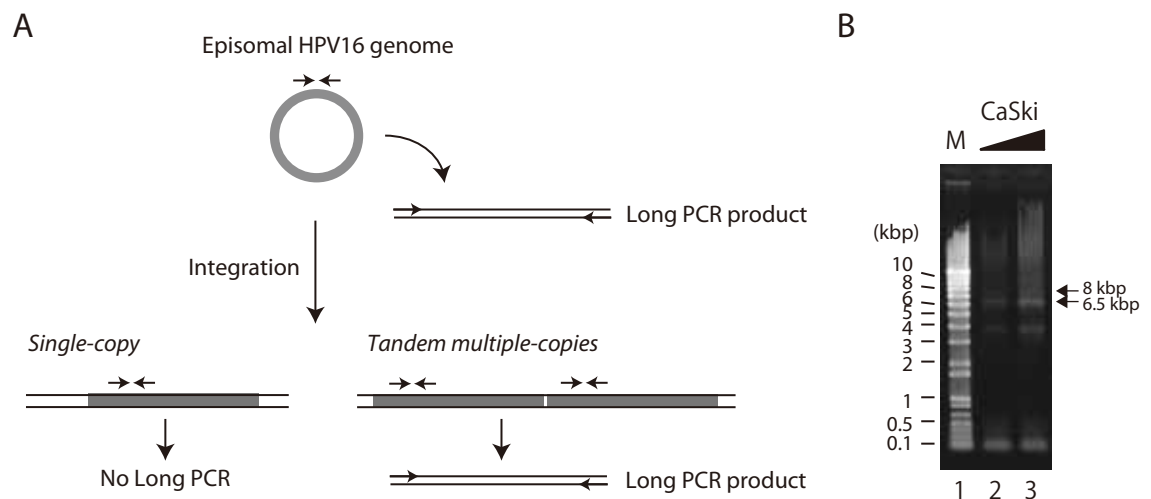

**Kukimoto et al. Fig. S2**

Supplement: Figure S2 — Amplification of full-length HPV16 genomes from CaSki cells. (A) Scheme for PCR amplification from integrated HPV16 genomes in cervical cancer. Tandem multiple-copies of integrated HPV16 DNA can be a template for full-circle PCR as well as episomal HPV16 genomes, while single-copy integrated HPV16 DNA cannot. (B) PCR was performed with primer-pair 1742F/1873R and increasing amounts of total DNA extracted from CaSki cells (lanes 2 and 3). M, DNA size marker (lane 1). (PDF) [file pone.0080583.s003.pdf]

A

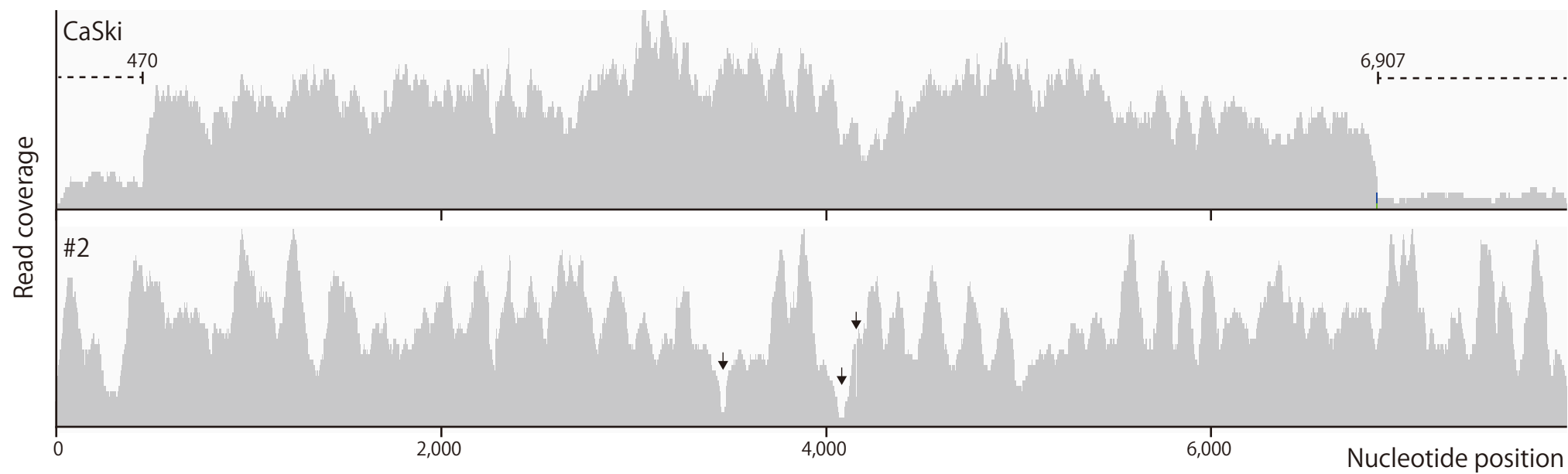

B

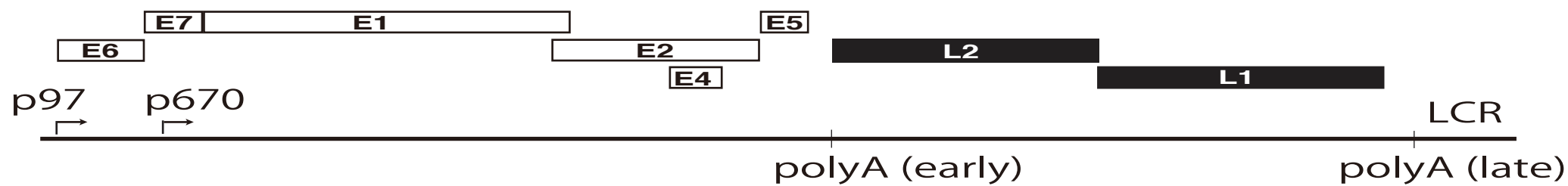

Kukimoto et al. Fig. S3

Supplement: Figure S3 — Read-depth profile obtained by deep sequencing of long PCR products. (A) Paired-end read sequences were aligned using BWA to each de novo assembled complete HPV16 genome sequence, and resultant read-depth profiles for CaSki and LSIL sample 2 are shown. Arrows indicate positions of the discontinuous distribution of read depth. The maximum read-depths were 30,173 for CaSki and 23,901 for sample 2. (B) Scheme for the genomic organization of HPV16. (PDF) [file pone.0080583.s004.pdf]

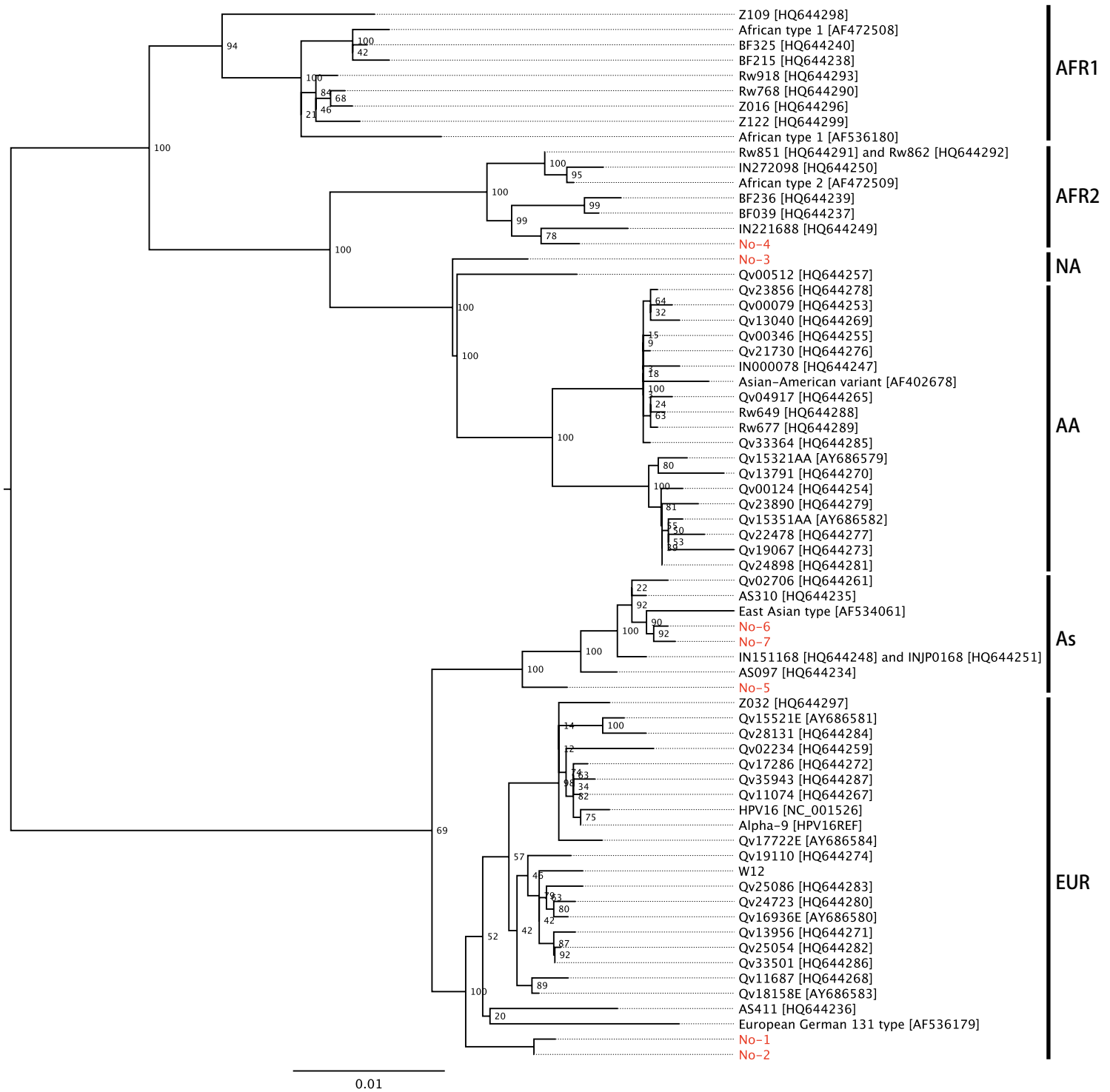

Kukimoto et al. Fig. S5

Supplement: Figure S5 — Phylogenetic analysis of denovo assembled complete HPV16 genome sequences. Sixty-two complete HPV16 genome sequences previously reported (Smith et al., PLoS One: 6, e21375, 2011) and 7 complete HPV16 genome sequences de novo assembled in this study were aligned against each other by mafft. Maximum likelihood phylogenetic tree was constructed using the aligned sequences with 1,000-fold bootstrapping. The scale indicates a branch length of 0.01 that shows 1% difference between the nucleotide sequences at the beginning and end of the branch. The number at each branch node represents the bootstrapping value. Vertical bars indicate major clades of HPV16 variants: AFR1, AFR2, NA, AA, As, and EUR. (PDF) [file pone.0080583.s006.pdf]

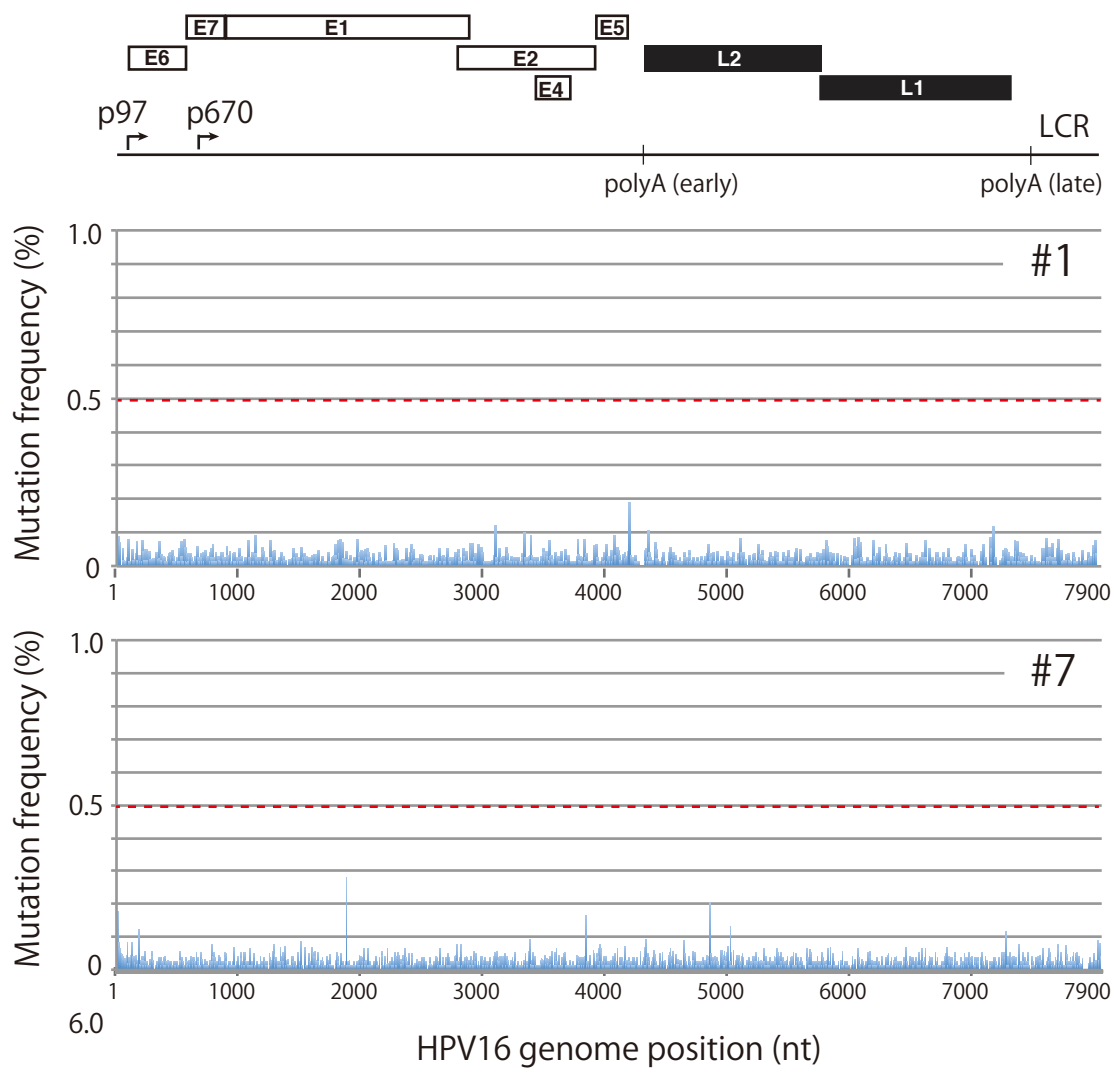

Kukimoto et al. Fig. S6

Supplement: Figure S6 — Mutation frequency profile of full-length HPV16 genomes in clinical specimens. The read sequences obtained with full-length HPV16 genomes prepared from clinical specimens (#1, LSIL; #7, ICC) were aligned to their de novo assembled complete genome sequences, and mutation/error frequencies at each nucleotide position are presented in the landscape of the full-length HPV16 genome. A threshold line for a reliable mutation frequency (0.5%) is indicated with the red dotted line. The genome organization of HPV16 is indicated above. (PDF) [file pone.0080583.s007.pdf]

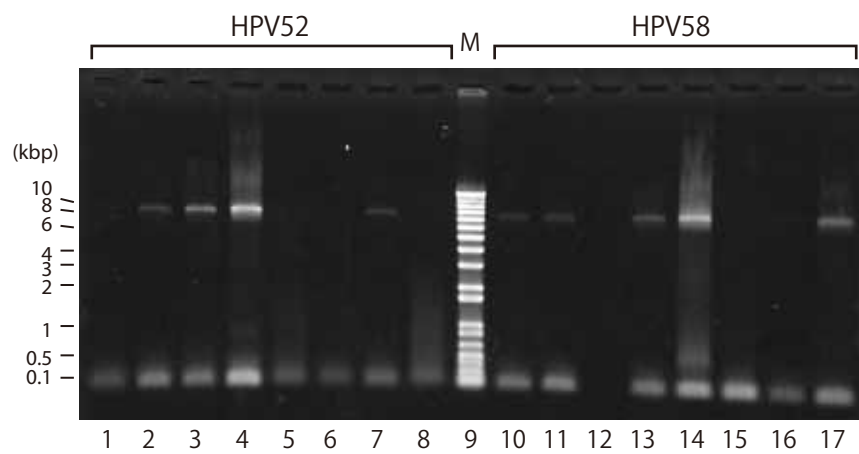

Kukimoto et al. Fig. S7

Supplement: Figure S7 — Amplification of full-length genomes of HPV52 and HPV58 from clinical specimens. Full-circle PCR was performed with HPV52 or HPV58-specific primers and DNA isolated from 8 HPV52-positive LSIL (lanes 1 to 8) and 8 HPV58-positive LSIL (lanes 10 to 17) specimens. Primers are as follows: HPV52-F, 5’-ACC AGA AAC ACA TAT GGT AAT AGA ACC-3’; HPV52-R, 5’-GTA ATA CTG TTT GTT GTT CTA TCC ATT C-3’; HPV58-F, 5’-TAC TAT CAA TTC CTG AAA CAT GTA TGA-3’; HPV58-R, 5’-AAT CTA TCT ATC CAT TCT GGT GTT G-3’. M, DNA size marker (lane 9). (PDF) [file pone.0080583.s008.pdf]

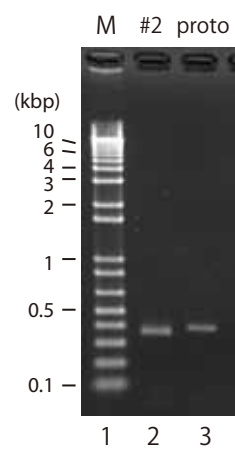

**Kukimoto et al. Fig. S8**

Supplement: Figure S8 — Amplification of deleted E2 sequence from a clinical specimen. Conventional PCR using AmpliTaq Gold was performed with HPV16-specific primers in the E2 gene and DNA extracted from LSIL specimen 2 (lane 2) and HPV16/pUC19 (lane 3). M, DNA size marker (lane 1). (PDF) [file pone.0080583.s009.pdf]
