# Supplementary material for: Genetic Variation of Human Papillomavirus Type 16 in Individual Clinical Specimens Revealed by Deep Sequencing
Source: PLoS One. 2013 Nov 13;8(11):e80583. doi: 10.1371/journal.pone.0080583 (PMC3827439; doi:10.1371/journal.pone.0080583)
Supplement: Figure S4 — Alignment of denovo assembled HPV16 genome sequences. Eight de novo assembled complete HPV16 genome sequences (samples 1 to 7, and W12) were aligned against each other by MAFFT. The sequence alignment around the non-coding region between E5 and L2 is presented. The stop codon of E5 is indicated with dotted-line box. Numbering of nucleotide positions is based on the sequence of #1. (PDF) [file pone.0080583.s005.pdf]

```
        4081      4091      4101      4111      4121      4131
#1      gcacgcttttaattacataatgtatatgtacataatgtaattgttacatataattgttg
#2      gcacgcttttaattacataatgtatatgtacataatgtaattgttacatataattgttg
#3      gcacgcttcttaattacataatgtatatgtacataatgtaattgttacatataattgttg
#4      gcacgcttcttaattacataatgtatatgtacataatgtaattgttacatataattgttg
#5      gcacgcttttaattacataatgtatatgtacataatgtaattgttacatataattgttg
#6      gcacgcttttaattacataatgtatatgtacataatgtaattgttacatataattgttg
#7      gcacgcttttaattacataatgtatatgtacataatgtaattgttacatataattgttg
W12     gcacgcttttaattacataatgtatatgtacaaaatgtaattgttacatataattgttg
        ********.************************ **************************

        4141      4151      4161      4171          4181         4191
#1      tataccataacttactattttttcttttttat-----ttttatatataa---tttttttt
#2      tataccataacttactattttttcttttttat-----ttttatatataattttttttttt
#3      tataccataaattactgatttttttttttttattttattttatatatag----ttttttt
#4      tataccatcacttactgatttttcttttgtat-----ttttatatatag---tttttttt
#5      tataccataacttactattttttcttttttat-----ttttatatatacttttttttttt
#6      tataccataacttactattttttcttttttat-----ttttatatataa-ttttttgttt
#7      tataccataacttactattttttcttttttat-----ttttatatataa-ttttttgttt
W12     tataccataacttactattttttcttttttat-----tttcatatatat--ttttttttt
        ******** * *****. *****.**** ***     ***.*******     *** ***
```

Kukimoto et al.   Fig. S4
